# Family coalitions and restrictive eating disorders in adolescence: a lausanne trilogue play study

**DOI:** 10.3389/fpsyg.2026.1833778

**Published:** 2026-06-03

**Authors:** Michela Criscuolo, Martina Maria Mensi, Ileana Croci, Ilenia Chianello, Arianna Vecchio, Chiara Coci, Marina Riso, Maria Chiara Castiglioni, James P. McHale, Valeria Zanna

**Affiliations:** 1 Anorexia Nervosa and Eating Disorder Unit, Child Neuropsychiatry, Department of Neuroscience, Bambino Gesù Children Hospital IRCSS, Rome, Italy; 2 Department of Brain and Behavioral Sciences, University of Pavia, Pavia, Italy; 3 Child Neuropsychiatry Unit, IRCCS Mondino Foundation, Pavia, Italy; 4 Predictive and Preventive Medicine Research Unit, Bambino Gesù Childrens’ Hospital IRCCS, Rome, Italy; 5 Family Study Center, University of South Florida, St. Petersburg, FL, United States

**Keywords:** adolescents, anorexia nervosa, coparenting, family coalitions, family relationships, family-based intervention, Lausanne Trilogue Play, restrictive eating disorders

## Abstract

**Background:**

Coparenting, defined as the ability of mothers and fathers to coordinate and provide mutual support in parenting, plays a key role in children’s social, emotional, and behavioral adjustment. While previous research has highlighted its influence on internalizing and externalizing problems, comparatively less is known about how children contribute to and respond to coparenting at a relational level.

**Objective:**

This study investigates coparenting behaviors in families of adolescents with restrictive eating disorders and examines their association with the patients’ interactive styles during the Lausanne Trilogue Play. We also explore potential correlations of coalition patterns with age, BMI, and clinical diagnosis.

**Methods:**

Seventy-seven adolescents with restrictive Eating Disorders (DSM-5) and their parents participated. Most families (81%) were intact. Coparenting coordination was assessed using the Coparenting and Family Rating System, while children’s interactive styles were coded using the Lausanne Trilogue Play reading grid adapted for preadolescents and adolescents.

**Results:**

Distinct coalition patterns emerged linking coparenting behaviors and adolescent interactive styles. Functional coparenting was associated with engaged child responses, whereas dysfunctional patterns corresponded to overinvolved responses. No significant correlations were found between coalition patterns and Body Mass Index, age, or specific Eating Disorders diagnosis, suggesting that these relational dynamics do not reflect current clinical status and needed more investigations.

**Conclusion:**

Identifying coalition patterns between coparenting and adolescent interactive styles provides valuable insights for family-based interventions in restrictive Eating Disorders. Such patterns allow clinicians to recognize both challenges and resources within the family system, informing psychoeducational and therapeutic strategies that integrate parental support with clinical and nutritional care.

## Introduction

Most of the world’s children grow up in family systems and collectives where multiple adults, or “coparents,” contribute to their care, socialization, acculturation, and sense of safety and belonging (McHale, 2007; McHale and Irace, 2011). The term coparenting refers to the mutual, joint efforts of coparents working together as a team to inspire in their members a sense of unity, camaraderie, and esprit de corps and promote children’s healthy social and emotional development (McHale et al., 2023). Coparenting is conceptualized as a triangular family process, at minimum, involving a child and at least two adults sharing responsibility for the care and upbringing of that child (McHale et al., 2023).

The coparenting relationship plays a pivotal role in children’s adjustment, parenting and family functioning. It is noted that coparenting influences the family’s emotional stability (Davies and Mark Cummings, 1994), parenting practices (Margolin et al., 2001; Bonds and Gondoli, 2007; Morrill et al., 2010), parental emotional availability (Sturge-Apple et al., 2006), and parent–child relationships (Feinberg and Kan, 2008; Teubert and Pinquart, 2010). Coparenting behavior also affects the children and adolescents’ adjustment, particularly with respect to social skills and functioning (Teubert and Pinquart, 2010). A meta-analysis of 93 studies involving 41,207 participants found that coparenting behavior was slightly and significantly related to children’s externalizing and internalizing problems (Zhao, 2022). A study of Kolak and Volling (2013) also highlighted the role of supportive coparenting in mediating emotional reactivity of the first child in the transition to siblinghood, constituting itself as a protective factor in the face of this transition for negatively reactive children.

The field of coparenting itself evolved from a theoretical foundation provided by Salvador Minuchin’s structural family theory (Minuchin, 1974). Minuchin’s work also catalyzed a significant line of inquiry tying coparenting and family processes to psychosomatic and health conditions, including Eating Disorders (Minuchin et al., 2009). In contemporary medical nomenclature, Restrictive Eating Disorders (RED) have come to be defined as a group of Eating Disorders (EDs) in which food restriction and selectivity prevail, with or without compensatory behaviors such as physical hyperactivity or self-induced vomiting. This group includes Anorexia Nervosa (AN), Atypical Anorexia Nervosa (A-AN) and Avoidant-Restrictive Food Intake Disorder (ARFID), because although they are very different in phenotype and diagnostic criteria, they are united by a significant and progressive weight loss due to food restriction or faltering growth, with consequent important implications for development (American Psychiatric Association, 2013).

EDs are among the most common mental illnesses in childhood and adolescence, after anxiety and depressive disorders (Polanczyk, 2015). AN, in particular, is the psychiatric illness with the highest mortality rate, mainly due to medical complications and suicide (Arcelus et al., 2011). A robust body of literature highlights how family functioning may be involved in the onset and maintenance of symptoms, as well as serve as a resource for treatment since the child or adolescent, living within the family context, both influences it and is, in turn, influenced by it (Holtom-Viesel and Allan, 2014; Del Casale et al., 2022). Relevant to the current investigation, several studies have highlighted, using observational and self-report tools, that families of patients with RED exhibit worse family functioning compared to controls or other categories of patients (Holtom-Viesel and Allan, 2014).

A largely used tool for family relations evaluations in the Eating Disorder research is the Lausanne Trilogue Play procedure (LTP) (Fivaz-Depeursinge and Corboz-Warnery, 1999; Malagoli Togliatti and Mazzoni, 2006), a semi-structured method for observing family dynamics, which enables analyses of adults’ and children’ s adherence to prescribed turn-taking roles, body formations and emotional sharing to characterize the three-person “family alliance” as functional or dysfunctional. Studies using the LTP standardized 4-Part find that families with a member with AN are more likely to exhibit dysfunctional alliances compared to non-clinical families or families with a member with internalizing problems (Balottin et al., 2017; Mensi et al., 2020). Such families tend to demonstrate strong physical and psychological participation but poor definition of roles and low emotional sharing (Balottin et al., 2017; Criscuolo et al., 2020). Low coordination was evidenced particularly in Parts 3 and 4 of the LTP, where parents are called to coordinate their parental interventions and to define clear spaces between themselves and their children’s subsystem.

Several studies (Tafà et al., 2019; Criscuolo et al., 2020; Criscuolo et al., 2020) have explicitly investigated coparenting dynamics in families with a member with AN. Results have shown that depressive symptoms and maladaptive imbalance in family functioning of adolescents with AN are influenced by contrasting dynamics between mothers and fathers (Tafà et al., 2019). Moreover, a presence of coparental conflict is associated with lower Body Mass Index (BMI) and higher dysfunctional family functioning (Criscuolo et al., 2020; Criscuolo et al., 2020). Using McHale’s coparenting categorization (McConnell and Kerig, 2002), analyses have identified a prevalence of “child-at-center” and “excluding” coparenting styles: in the first, children’s preferences and initiatives dictate the flow of interaction in the family, with parents largely reactive rather than offering guidance and direction to the child, sometimes to the point of establishing a role reversal dynamic (Kerig, 2005). The second one indicates a pattern in which one parent shows a greater involvement in the child than the other, excluding the second coparent.

Based on intriguing evidence concerning children’s predisposition to communicate at a triadic level (collective intersubjectivity), Fivaz-Depeursinge and colleagues (Fivaz-Depeursinge et al., 2009) highlighted that the child is seldom neutral in response to coparenting behaviors, but typically responds in ways that can, in turn, influence these behaviors. The authors distinguished the child’s contributions into three specific “interactive styles”: *engaged, disengaged, and overinvolved*. The *engaged child* is involved in interaction with the parents—also emotionally—and is ready to follow their directions, showing a desire to share the experience with both. The *disengaged child* disconnects from the parents and interacts with them primarily through the mediation of objects, often conforming excessively to parental requests. The *overinvolved interactive style* indicates a child who uses confrontation and/or animation tactics in manipulative and controlling ways that are not age appropriate. The findings demonstrated that children begin to self-regulate early on, adapting to a wide range of conditions proposed by the parents, and that these interactive exchanges can be operationalized according to the coalition models originally proposed by Minuchin and colleagues (Minuchin et al., 1978): (1) *detouring*, whereby apparently united parents cover up their conflicts, assuming a protective position toward the sick child or blaming him or her; (2) *triangulation*, in which the patient is called by parents to side against the other; and (3) *binding*, in which the child is permanently united with one parent against the other. The child’s interactive style and coalition patterns are associated with greater difficulties both in terms of the child’s psychopathological functioning and in the levels of family coordination and overall functioning (Fivaz-Depeursinge et al., 2009; Mazzoni et al., 2015).

Given these important precedents in the research literature, one compelling set of questions concerns how a patient with RED contributes to the coparenting dynamic presented by the parents, and whether particular responses might be associated with specific coparenting styles. Should it be possible to identify coherent interactive patterns, the assessment of these patterns can facilitate a more precise definition of forms of family dysfunction. Optimally, such an understanding could lead to the identification of customized clinical intervention methods that can assist parenting and coparenting, ranging from psychoeducation to psychotherapy. This study hence aims to examine coparenting behaviors in families of patients with RED and to trace their association with the patients’ interactive style during the clinical LTP (Fivaz-Depeursinge and Corboz-Warnery, 1999; Malagoli Togliatti and Mazzoni, 2006). We will also investigate if specific coalitions correlate to the patient’s diagnosis, BMI or age at the assessment.

## Methods

### Participants

The sample is composed of 77 adolescents with restrictive ED according to DSM-5 and their parents, recruited at Bambino Gesù Children Hospital in Rome and Fondazione Mondino in Pavia, specialized in EDs evaluation and treatment. Each family was asked to take part in the standardized LTPc procedure. The play was videotaped and then coded by clinicians trained in coparenting coding. Coparenting was measured using the Coparenting and Family Rating System (CFRS) (McConnell and Kerig, 2002; McHale et al., 2000), while the reading grid proposed by Fivaz Depeursinge and adapted to pre-adolescent and adolescent children was used to measure the children’s interactive styles (Fivaz-Depeursinge et al., 2009; Mazzoni et al., 2015).

### Measures

#### Lausanne Trilogue Play

The LTP is a standardized and well-validated observation-based method used in clinical and research settings to assess dysfunctional patterns in triadic or family interactions (Malagoli Togliatti and Mazzoni, 2006). The procedure requires parents and adolescent to sit around a table and to write a story about a weekend without the parents. The play is divided into four phases. In the first two parts, parents take turns interacting with the adolescent while the other one observes; in the third part, both parents are active with their child and help him or her write the story. Finally, in the fourth part, the adolescent plays alone, while the parents have their own discussion. The entire process is videotaped and lasts ∼15 min.

The LTP coding system used in this study has been explained in previous publications (Lavadera et al., 2011; Mazzoni et al., 2015; Balottin et al., 2017). Essentially, it considers four aspects of interaction (i.e., participation, organization, focal attention, affective contact), which are rated, in each phase, on a three-point Likert scale (0 = dysfunctional, 1 = partially functional, 2 = functional). On this basis, descriptions of each family member’s interactive contribution and of the overall family functioning are obtained. The total family score, which identifies one of four types of family alliance, is the sum of the scores recorded by each family member in each phase.

#### Coparenting and family rating system

Coparenting was evaluated using the Coparenting and Family Rating System—Toddler age and above (CFRS) (McConnell and Kerig, 2002; McHale et al., 2000, 2004) during the LTP procedure. The CFRS is a coding system that assesses coparenting dynamics through an analysis of interactive patterns, focusing on the degree of cooperation and competition in the parental couple, investment and warmth toward the child and between parents, child- (as opposed to parent-) centeredness, and the presence of verbal sparring between parents. Scores range from 1 (absence) to 5 (elevated presence) for all scales, except for “Investment and Warmth Toward the Child” scale, which range from 1 to 7. The coding is based on video observations of the family interaction during the Part 3 of the LTP by two independent and reliable judges, who are blind to the goals of the research. The judges assign scores to each scale and then determine the overall coparenting style. The CFRS was originally developed for families with infants and preschool-age children, but it has also been used with families of school-age children (McConnell and Kerig, 2002; Fivaz-Depeursinge et al., 2009) and adolescents (Mazzoni et al., 2015).

Analyses of data from CFRS studies identified four family patterns with distinctive coparenting dynamics: cohesive, excluding, competitive, and child-at-center (McHale et al., 2002). The *cohesive style* is characterized by balanced levels of parenting involvement with the child, high levels of warmth both from parents to child and between parents, high coparental cooperation and low levels of competition. These families harmonize well, both within the inter-parental relationship and within each parent–child relationship. The *child-at-the-center style* is characterized by an almost exclusive focus on the child by both parents accompanied by a noteworthy disconnection between the coparents themselves. In the *competitive coparenting style*, parents are equally involved with their child but interactions are characterized by competition between one another and a lack of warmth and cohesion. Finally, in the *excluding style*, parents are disconnected from each other and there is a large discrepancy in each partner’s level of involvement with the child, such that one parent is largely disengaged from the interaction. The authors also identified a shifting coparenting style, in which a shift from a functional to a dysfunctional style was demonstrated in the same interaction.

#### Children’s interactive styles

The Lausanne Group developed a coding system characterizing the child’s behavior within the context of the LTP (Fivaz-Depeursinge et al., 2009). Because Fivaz’s coding system was developed for and was most applicable to children aged 0 to 3 years, some adaptations were needed when examining parallel behaviors of older children in a triadic context. For the present study, an integration proposed by Mazzoni and colleagues (Mazzoni et al., 2015) was utilized. Their adaptation incorporated a variety of behavioral indicators that were more suitable for older children and adolescents, emphasizing their more developed social skills (compared to children in the sample considered by Fivaz-Depeursinge). Highlights of the coding manual for defining children’s interactive styles are described in Table 1.

**Table 1:** Children interactive styles according to Mazzoni et al. (2015) classification.

Children/Adolescent interactive styles	Behavioral indicators
Engaged	A willing and active child, who addresses the active parent or both parents depending on the different parts of the game. Furthermore, he or she consistently expresses—through gaze, verbalizations, and actions—attention to the ongoing activity and the contributions of others.
Disengaged	A child who shows no interest in the activity at hand, isolates himself and does not cooperate with his parents, but plays alone.
Overinvolved	Child is extremely direct in his interactions with his parents: he guides the game toward self-defined goals, is oppositional, and refuses to accept the role of third party during Part IV of the game, perceiving it as exclusion.
Shifting	Shifts between functional and dysfunctional styles throughout the process.

#### BMI percentile

Body Mass Index percentile (BMIp) is an age- and sex-specific measure used primarily for children and adolescents to assess body weight. It identifies four standard weight status categories (Centers for Disease Control and Prevention guidelines): underweight (< 5th percentile), healthy weight (from 5th to < 85th percentile), overweight (from 85th to < 95th percentile) and obesity (≥ 95th percentile).

Although the 5th BMIp is the weight cut-off criterion in the Diagnostic and Statistical Manual of Mental Disorders-5 (DSM-5) for diagnosing AN in children and adolescents, its validity has not been proven and the 10th percentile value is often applied. A recent study (Monteleone et al., 2001) have noted that the 5th BMI percentile does not discriminate psychopathology severity in adolescents with AN so in this study the 10th percentile is used to discriminate the sample in two categories.

### Statistical analysis

Data are presented as counts and percentages for categorical variables, and as median with interquartile range (IQR) for continuous variables. Chi-square goodness-of-fit tests were performed to evaluate whether the distribution of categorical variables (e.g., coparenting patterns and interactive styles) significantly differed from a uniform distribution. Associations with age, diagnosis and BMIp were investigated using Chi-square test. Relationships between coparenting patterns and interactive styles were further explored using chi-square tests and multinomial logistic regression models, both unadjusted and adjusted for relevant clinical and demographic covariates (data not shown). Results were considered statistically significant at *p* < 0.05. All analyses were conducted using STATA version 18.0 (StataCorp, College Station, TX, United States).

## Results

Descriptive data are provided in Table 2 for the 77 index patient study participants, all Caucasian females being cared for in day-hospital settings at the time of their participation. All 77 took part in the study with their parents.

**Table 2:** Characteristics of the sample (*n* = 77).

	*n*	%
Patients’ characteristics		
Age (median, IQR)	15 (13.8–16.3)	
Age in classes		
11–13	21	27.3
14–18	56	72.7
Females	73	94.8
BMI (median, IQR)	15.8 (14.5–17.0)	
BMI percentile		
<10 percentile	50	64.9
10–85 percentile	27	35.1
Comorbidities	41	53.9
Siblings		
No	19	24.7
1 Sibling	41	53.2
2 Siblings or more	17	22.1
Birth order		
Twins	3	3.9
Firstborn	38	49.4
Second-born	31	40.3
Third-born or later	5	6.5
Diagnosis		
Typical AN	45	58.4
Atypical AN	19	24.7
ED NAS	13	16.9
Families’ characteristics		
Type of families		
United family	63	81.2
Separated family	12	15.6
Blended family	2	2.6
Mother’s age (median, IQR)	48 (44–53)	
Mother’s education		
Secondary school certificate	12	17.1
High school certificate	35	50.0
University degree	23	32.9
Father’s age (median, IQR)	51 (47–55)	
Father’s education		
Secondary school certificate	15	21.4
High school certificate	32	45.7
University degree	23	32.9

IQR, Interquantile Range; BMI, Body Mass Index; AN, Anorexia Nervosa; ED NAS, Eating Disorders Not Otherwise Specified.

Patients’ median age was 15 years old, with a prevalent diagnosis of typical AN and a BMIp under the 10th percentile. Families were united (81%) with median age of mothers 48 years old and median age of fathers 51 years old. High school certificates had been obtained by 45–50% of parents, with another third of parents having obtained a university degree.

Tables 3, 4 show prevalence of coparenting and patients’ interactive styles, stratified for age, BMI and diagnosis. 11.7% of parents did not play the LTP Part 3, so neither coparenting style nor patients’ interactive style could be coded. No differences in prevalence were found between groups for coparenting and for adolescents’ interactive styles. However, the goodness of fit was significant for *p* = 0.035 for coparenting distribution and for *p* = 0.003 for interactive styles distribution. So, in this sample most coparenting styles were unbalanced, with primarily child-at-center and excluding patterns, and most patients showed overinvolved interactive styles.

**Table 3:** Coparenting stratification for BMI, age and diagnosis.

Coparenting		Percentile BMI		*p*	Age		Diagnosis				*p*
	Total (%)	≤10°(%)	>10°(%)		11–13(%)	14–18 (%)	*p*	AN (%)	A-AN (%)	NAS (%)	
Cohesive	14.7	11.4	20.8	0.809	14.3	12.5	0.554	6.7	15.8	30.8	0.421
Excluding	32.4	36.4	25.0		23.8	30.4		35.6	26.3	7.7	
Competitive	20.6	20.5	20.8		9.5	21.4		17.8	15.8	23.1	
Child-centered	32.4	31.8	33.3		33.3	26.8		26.7	31.6	30.8	
No part 3	11.7	12	11.1		19.1	8.9		13.3	10.5	7.7	

BMI, Body Mass Index.

**Table 4:** Patients’ interactive styles stratification for BMI, age and diagnosis.

Patience interactive style		Percentile BMI		*p*	Age		Diagnosis				*p*
	Total	≤10°	>10°		11–13	14–18		AN	A-AN	NAS	
	*n* (%)	*n* (%)	*n* (%)		*n* (%)	*n* (%)	*p*	*n* (%)	*n* (%)	*n* (%)	
Engaged	18.8	20.5	16.0	0.667	16.7	19.6	0.871	18.0	11.1	4 33.3	0.534
Disengaged	21.7	18.2	28.0		16.7	23.5		20.5	33.3	8.3	
Overinvolved	42.0	40.9	44.0		44.4	41.2		41.0	38.9	50.0	
Shifting	17.4	20.5	12.0		22.2	15.7		20.5	16.7	8.3	

BMI, Body Mass Index.

### Patterns of coalition

**Figure 1:**
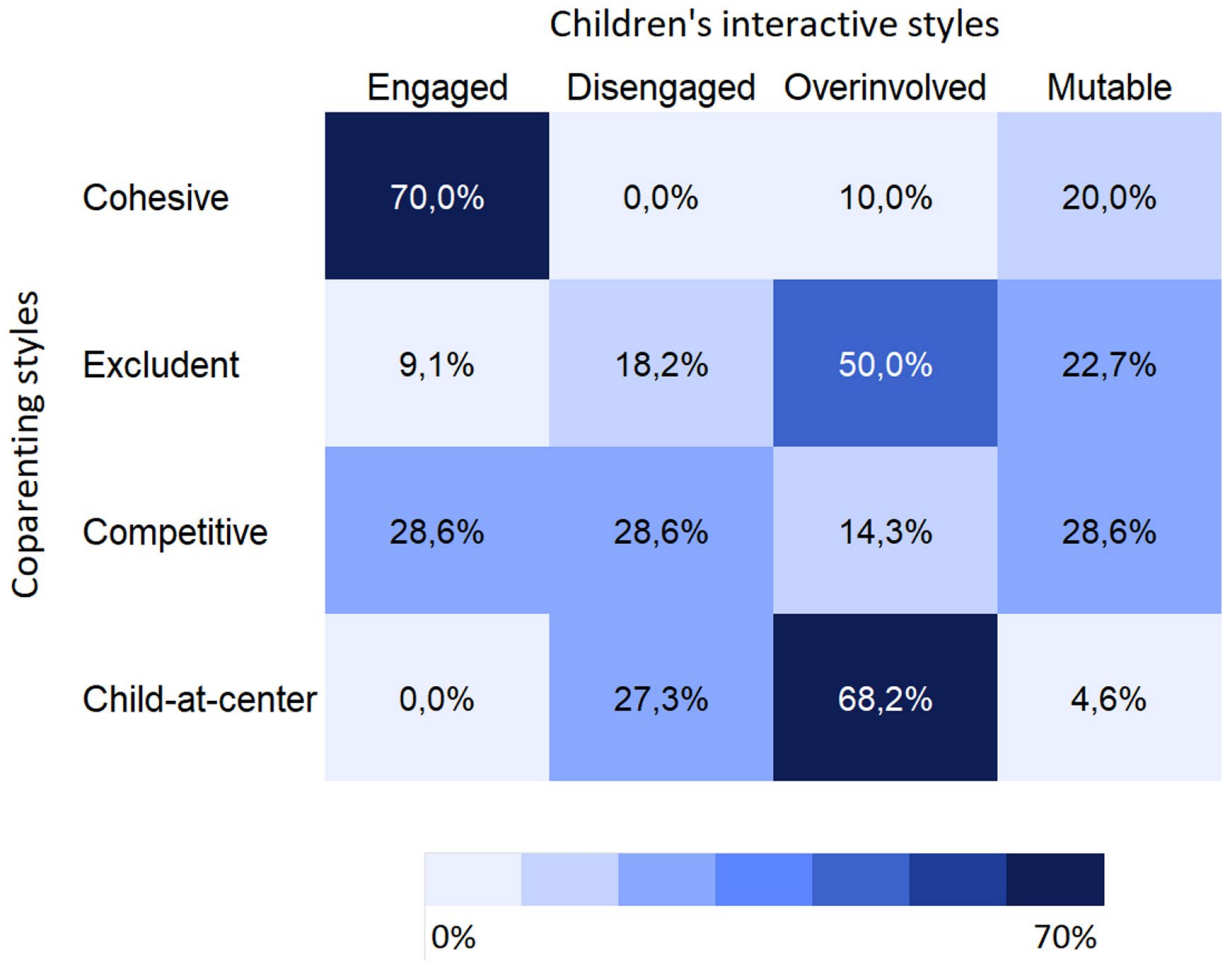
Heatmap of association between coparenting and patience interactive styles.

Figure 1 shows a heatmap portraying the association between coparenting and patients’ interactive styles. When overlapping, we used the operationalization model of triadic configurations already highlighted by Fivaz-Depeursinge et al. (2009) to name the coparenting-child response interactive patterns. In our sample there was a strong relationship between cohesive coparenting and the engaged interactive style, reflecting a positive alliance between parents and adolescent. In the same way, analysis shows strong evidence also for two negative coalitions: child-at-center coparenting - overinvolved interactive style (detouring) and excluding coparenting-overinvolved interactive style (binding).

No clear association was found for competitive coparenting which showed associations both with engaged and disengaged (triangulation) as well as with shifting interactive styles.

Patterns of coalitions generally did not vary as a function of BMI, age or diagnosis (Table 5).

## Discussion

RED, a category of EDs with age of onset typically in preadolescence or adolescence, are associated with elevated mortality rates (Arcelus et al., 2011). The role of parents and family relationships has been extensively investigated in the ED literature, both as a potential maintaining factor of disordered eating symptoms and as a resource for positive clinical outcomes (Liebman et al., 1974; Russell, 1987; Lock et al., 2000; le Grange et al., 2010). By contrast, comparatively less attention has been devoted to examining the relational contributions of the patient to the triadic interactions in which they participate.

Several studies have emphasized that, within parent–child triadic relationships, the child is never a neutral participant; rather, through their responses and triadic bids, they actively contribute to family coordination and coparenting processes (Fivaz-Depeursinge and Corboz-Warnery, 1999; Fivaz-Depeursinge et al., 2009). Coparenting quality—particularly, parents’ shared engagement and teamwork, their capacity to collaborate effectively toward common goals and to provide mutual support—may represent a critical focus for the treatment of restrictive ED in developmental populations. Within a psychoeducational framework that operates in integration with other healthcare professionals, interventions may target parents’ ability to support and implement the nutritional guidelines provided by clinicians. However, within an interconnected system of interactions and communicative exchanges, the quality of the patient’s responses to coparenting dynamics must also be considered as they also appear to play a significant role in shaping the interactions among family members. Consequently, they may also influence treatment response. Prior studies with CFRS had already documented a higher prevalence of unbalanced coparenting patterns in samples of patients with RED (Criscuolo et al., 2020; Criscuolo et al., 2020), most commonly “child-at-center” and “excluding” dynamics. The defining feature of the former is a shared focus on the child in the absence of genuine coordination between parents, whereas the latter describes a pattern in which one coparent displays more intensive involvement with the child, often effectively marginalizing the other coparent.

The present study sought to investigate through CFRS the potential association between the quality of coparenting interactive exchanges and the interactive responses of offspring with RED, to determine whether it was possible to establish coherent patterns of communication and relational exchange. The use of CFRS to adolescence, while requiring further replication, represents a meaningful contribution to the literature, as its dimensions—grounded in observable behaviors and minimally reliant on interpretation—capture broad coparenting processes that are not age-specific and remain salient across development. However, in the absence of a control or comparison group, these findings should be interpreted as descriptive in nature rather than as specific to RED. Study findings indicate a greater likelihood that balanced coparenting styles are associated with balanced interactive responses in the child (cohesive engaged), whereas unbalanced coparenting styles are linked to unbalanced interactive responses, and vice versa.

Consistent with previous work (Fivaz-Depeursinge et al., 2009; Mazzoni et al., 2015), our sample revealed significant associations between cohesive coparenting and an engaged adolescent’s interactive style; between child-at-center coparenting and overinvolved adolescent’s responses; and between excluding coparenting and overinvolved interactive styles. In contrast, competitive coparenting did not show a specific association with a single adolescent response style. Rather, adolescents with RED in this category engaged in multiple, different patterns or family dynamics, including cohesive, disengaged and shifting. Previous research conducted on both clinical and non-clinical samples (Mazzoni et al., 2015) found that children from separated families characterized by high interparental conflict and referred by court-appointed consultants more frequently exhibited an engaged (i.e., functional) interactive style compared with families undergoing psychotherapy for internalizing or externalizing child difficulties (Mazzoni et al., 2015). These findings, while open to interpretation, may suggest that, under certain circumstances, children can draw upon personal or social resources to enact more adaptively during interactions. Alternately, because competitive coparenting (characterized by overt or covert conflictual dynamics) involve both parents vying for the child’s attention and for leadership of the ongoing activity, unexamined moderating factors may evoke or sustain more functional exchanges than those observed in other coalition configurations identified in the present study.

Parent–child coalition models were originally conceptualized by Fivaz-Depeursinge et al. (2009), drawing on coparenting styles described by McHale et al. (2002) and on the classification of children’s interactive styles developed by the Lausanne Group. Authors proposed three types of family coalitions corresponding to the triadic interaction models identified by Minuchin (1974) in his work with families: binding/coalition, triangulation, and detouring. The sample examined by Fivaz-Depeursinge predominantly included school-aged children, and the presence of such coalitions had not yet been extensively explored in preadolescent and adolescent populations. The present study appears to support the validity of coalition models—operationalized through the coding of coparenting and adolescents interactive styles—also in developmental stages beyond middle childhood.

The absence of Part 3 of the LTP in 11.7% of the sample is clinically meaningful and should be considered an index of a dyadic relational style and reduced family-level integration rather than missing procedural data. This phase of the LTP specifically assesses the family’s capacity for shared, integrative functioning; thus, its omission may reflect a structural limitation in family organization, characterized by a predominance of dyadic exchanges and reduced flexibility in transitioning to triadic configurations. Evidence consistently indicates that families of individuals with RED, particularly AN, show marked difficulties in engaging in triadic interactions requiring coordinated participation of all members, particularly in terms of affect regulation, parental coordination, and limited triadic integration (Mensi et al., 2020; Balottin et al., 2017; Criscuolo et al., 2020).

Finally, we examined whether coalition patterns were associated with adolescents’ social and relational competencies, potentially linked to age or clinical status. The results did not reveal significant connections between coalition models and patient age, clinical presentation (in terms of differential diagnosis within RED), or BMI percentile. These findings suggest that coalition patterns may not be directly related to the current clinical condition, but rather to relational modalities that warrant further longitudinal investigation, given the cross-sectional nature of the study.

## Limitations and clinical implications

One of the primary limitations of the present study is the absence of a control group comprising healthy preadolescents and adolescents or youths with other clinical, gender or social conditions. The inclusion of such comparison groups would have enabled a clearer understanding of whether the coalition patterns identified are specific to ED or are similarly distributed across other clinical and non-clinical populations. A further limitation concerns the assessment of coparenting quality and the patient’s interactive style within a single observational session. The reliance on a single observational session further limits the ability to generalize findings to broader family functioning, as this methodological choice may have restricted the range of observable family behaviors and, consequently, limited the possibility of obtaining a coding truly representative of the family’s everyday relational dynamics.

Additionally, approximately 58% of the girls in the study had one or more siblings. Future research should examine not only the responses of the identified patient but also those of siblings within the family context, to determine whether coparenting patterns and interactive styles vary across family members or instead display steadiness and rigidity. From a triangular perspective, each sibling in the family is embedded in a distinctive mother–father-child coparenting dynamic, at the same time as family-wide coparenting patterns also exist. Several studies have also highlighted that siblings occupy a privileged position as third-party observers of the illness, while simultaneously expressing a need for greater understanding of their brother’s or sister’s condition and increased involvement in care contexts (Dimitropoulos et al., 2009; Hutchison et al., 2022). Further exploration of their role within dyadic, triadic and “n-adic” (more than three; Ricci and Selvini-Palazzoli, 1984) relational configurations may facilitate their inclusion and represent an important resource for the treatment of restrictive eating disorders in developmental populations.

Finally, a consideration about the context in which the sample was recruited. Families were accessed through two clinical services in Italy with a specific emphasis on parental involvement in treatment. This may have influenced the representation of parenting and coparenting styles, as families who voluntarily access such services may be more likely to present with relatively cohesive and/or child-centered interaction patterns.

Over time, the use of the Lausanne Trilogue Play (LTP) in both clinical and research settings has underscored the value of a structured observational tool capable of assessing relational competencies at multiple levels: individual members, specific subsystems, and the family as an integrated whole. Identifying coalition patterns and, more specifically, the reciprocity between coparenting style and child interactive style enables clinicians to work—through video-feedback interventions (Philipp et al., 2026)—on the specific strengths and vulnerabilities of each family, tailoring psychoeducational and psychotherapeutic techniques in integration with nutritional intervention and clinical monitoring.

**Table 5:** Coalitions stratification for BMI, age and diagnosis.

Coalitions	Percentile BMI		*p*	Age		Diagnosis				*p*
	≤10°(%)	>10° (%)		11–13 (%)	14–18 (%)	*p*	AN (%)	A-AN (%)	NAS(%)	
Alliance	14.8	16.7	0.992	16.7	15.2	0.764	13.0	16.7	20.0	0.947
Binding	25.9	22.2		33.3	21.2		30.4	25.0	10.0	
Detouring	33.3	33.3		33.3	33.3		30.4	33.3	40.0	
Triangulation	25.9	27.8		16.7	30.3		26.1	25.0	30.0	

BMI, Body Mass Index.

Parental involvement in eating disorders during developmental stages—particularly family-based interventions—is widely regarded as the gold standard of treatment (National Institute for Health and Care Excellence, 2020; Hay et al., 2023; Crone et al., 2025). However, the manner in which such interventions should be adapted to the specific relational configurations of individual families remains insufficiently explored. In this regard, the current study offers numerous promising leads. For example, one coalition observed in our sample, characterized by competitive coparenting and a mutable child response style, points to potentially valuable systemic resources that could be strengthened. Within family sessions, psychoeducational interventions could focus on enhancing coparental coordination by building upon the parents’ high levels of attention toward the child and leveraging functional aspects of the dyadic parent–child relationships (mother–child, father–child). Similarly, a detouring coalition might be addressed through family sessions integrated with nutritional treatment spaces, in which the patient is supported in moving beyond role-reversal or disengagement dynamics. Concurrently, parents may be offered a therapeutic space to gradually acknowledge and address couple-level conflict, thereby constructing a climate of trust in which such difficulties can be processed without involving the child and facilitating the reactivation of an age-appropriate individuation process.

These examples are of course speculative but reflect the kind of specificity that may be possible following further concerted study of family patterns and child participation in the manner explored in this study. Such work promises to provide an evidentiary basis that might significantly enhance treatment options for families with children suffering from RED.

## Data Availability

The raw data supporting the conclusions of this article will be made available by the authors, without undue reservation.

## References

[ref1] American Psychiatric Association. (2013). Diagnostic and statistical manual of mental disorders (5th ed.). Washington, DC: Author.

[ref2] Arcelus J. Mitchell A. J. Wales J. Nielsen S. (2011). Mortality rates in patients with anorexia nervosa and other eating disorders: a meta-analysis of 36 studies. Arch. Gen. Psychiatry 68, 724–731. doi: 10.1001/archgenpsychiatry.2011.74, 21727255

[ref3] Balottin L. Mannarini S. Mensi M. M. Chiappedi M. Gatta M. (2017). Triadic interactions in families of adolescents with anorexia nervosa and families of adolescents with internalizing disorders. Front. Psychol. 7, 1–13. doi: 10.3389/fpsyg.2016.02046, 28119647 PMC5221675

[ref4] Bonds D. D. Gondoli D. M. (2007). Examining the process by which marital adjustment affects maternal warmth: the role of Coparenting support as a mediator. J. Fam. Psychol. 21, 288–296. doi: 10.1037/0893-3200.21.2.288, 17605551

[ref5] Criscuolo M. Laghi F. Mazzoni S. Castiglioni M. C. Vicari S. Zanna V. (2020). How do families of adolescents with anorexia nervosa coordinate parenting? J. Child Fam. Stud. 29, 2542–2551. doi: 10.1007/s10826-020-01740-2

[ref6] Criscuolo M. Marchetto C. Chianello I. Cereser L. Castiglioni M. C. Salvo P. . (2020). Family functioning, coparenting, and parents’ ability to manage conflict in adolescent anorexia nervosa subtypes. Fam. Syst. Health 38, 151–161. doi: 10.1037/fsh0000483, 32525350

[ref7] Crone C. Fochtmann L. J. Ahmed I. Balas M. C. Boland R. Escobar J. I. . (2025). The American Psychiatric Association practice guideline for the prevention and treatment of delirium. Am. J. Psychiatry 182, 880–884. doi: 10.1176/appi.ajp.25182013, 40887950

[ref8] Davies P. T. Mark Cummings E. (1994) Marital conflict and child adjustment: an emotional security hypothesis Psychol. Bull. American Psychological Association Inc. 116: 387–411. doi: 10.1037/0033-2909.116.3.387, 7809306

[ref9] Del Casale A. Adriani B. Modesti M. N. Virzì S. Parmigiani G. Vento A. E. . (2022). Anorexia nervosa and familial risk factors: a systematic review of the literature. Curr. Psychol. 42, 25476–25484. doi: 10.1007/s12144-022-03563-4

[ref10] Dimitropoulos G. Klopfer K. Lazar L. Schacter R. (2009). Caring for a sibling with anorexia nervosa: a qualitative study. Eur. Eat. Disord. Rev. 17, 350–365. doi: 10.1002/erv.937, 19585664

[ref11] Feinberg M. E. Kan M. L. (2008). Establishing family foundations: intervention effects on Coparenting, parent/infant well-being, and parent-child relations. J. Fam. Psychol. 22, 253–263. doi: 10.1037/0893-3200.22.2.253, 18410212 PMC3178882

[ref12] Fivaz-Depeursinge E. Corboz-Warnery A. (1999). The Primary Triangle: A Developmental Systems View of Mothers, Fathers and Infants. New York, NY: Basic Books.

[ref13] Fivaz-Depeursinge E. Lopes F. R. A. N. C. E. S. C. O. Python M. A. R. Y. L. I. N. E. Favez N. I. C. O. L. A. S. (2009). Coparenting and toddler’s interactive styles in family coalitions. Fam. Process 48, 500–516. doi: 10.1111/j.1545-5300.2009.01298.x, 19930435

[ref14] Hay P. J. Rankin R. Ramjan L. Conti J. (2023). ‘Current approaches in the recognition and management of eating disorders’, the medical journal of Australia. John Wiley Sons Inc 219, 127–134. doi: 10.5694/mja2.52008, 37356068

[ref15] Holtom-Viesel A. Allan S. (2014). A systematic review of the literature on family functioning across all eating disorder diagnoses in comparison to control families. Clin. Psychol. Rev.. Elsevier Ltd 34, 29–43. doi: 10.1016/j.cpr.2013.10.005 24321132

[ref16] Hutchison S. House J. McDermott B. Simic M. Baudinet J. Eisler I. (2022). Silent witnesses: the experience of having a sibling with anorexia nervosa. J. Eat. Disord. 10:134. doi: 10.1186/s40337-022-00655-1, 36068560 PMC9450355

[ref18] Kerig P. K. (2005). Revisiting the construct of boundary dissolution: a multidimensional perspective. J. Emot. Abus. 5, 5–42. doi: 10.1300/J135v05n02_02

[ref19] Kolak A. M. Volling B. L. (2013). Coparenting moderates the association between firstborn children’s temperament and problem behavior across the transition to siblinghood. J. Fam. Psychol. 27, 355–364. doi: 10.1037/a0032864, 23750518 PMC3698979

[ref20] Lavadera A. L. Laghi F. Togliatti M. M. (2011). Assessing family coordination in divorced families. Am. J. Fam. Ther. 39, 277–291. doi: 10.1080/01926187.2010.539479

[ref21] le Grange D. Lock J. Loeb K. Nicholls D. (2010). Academy for eating disorders position paper: the role of the family in eating disorders. Int. J. Eat. Disord. 43, 1–5. doi: 10.1002/eat.20751, 19728372

[ref22] Liebman R. Minuchin S. Baker L. (1974). The role of the family in the treatment of anorexia nervosa. J. Am. Acad. Child Psychiatry 13, 264–274. doi: 10.1016/S0002-7138(09)61315-7, 4826071

[ref23] Lock J. le Grange D. Agras W. S. Dare C. (2000). Treatment Manual for Anorexia Nervosa. A Family Based Approach. New York, London: Guilford Press.

[ref24] Malagoli Togliatti M. Mazzoni S. (2006). Osservare, Valutare, e Sostenere la Relazione Genitori-Figli. Il Lausanne Trilogue Play Clinico [Observe, Evaluate, and Support the Parent-Child Relationship. The Clinical Lausanne Trilogue Play]. Milano: Raffaello Cortina Editore.

[ref25] Margolin G. Gordis E. B. John R. S. (2001) Coparenting: a link between marital conflict and parenting in two-parent families J. Fam. Psychol. American Psychological Association Inc. 15: 3–21. doi: 10.1037/0893-3200.15.1.3 11322083

[ref26] Maria Monteleone A. Mereu A. Cascino G. Ruzzi V. Castiglioni M. C. Patriciello G. . (2020). The validity of the fifth and the 10th body mass index percentile as weight cut-offs for anorexia nervosa in adolescence: no evidence from quantitative and network investigation of psychopathology. Eur. Eat. Disord. Rev. 29, 232–244. doi: 10.1002/erv.2814, 33314419

[ref27] Mazzoni S. Lavadera A. L. Di Benedetto R. Criscuolo M. Mangano C. (2015). Parenting coalitions: coparenting and toddler’s interactive styles. Societa Editrice Il Mulino 19, 79–100. doi: 10.1449/79740

[ref28] McConnell M. C. Kerig P. K. (2002) Assessing coparenting in families of school-age children: validation of the Coparenting and family rating system Can. J. Behav. Sci. Canadian Psychological Association 34: 44–58. doi: 10.1037/h0087154

[ref29] McHale J. P. (2007). When infants grow up in multiperson relationship systems. Infant Ment. Health J. 28, 370–392. doi: 10.1002/imhj.20142, 21512615 PMC3079566

[ref30] McHale J. P. Irace K. (2011). “Coparenting in diverse family systems,” in Coparenting: A Conceptual and Clinical Examination of Family Systems, (Washington, DC: American Psychological Association), 15–37.

[ref31] McHale J. P. Kazali C. Rotman T. Talbot J. Carleton M. Lieberson R. (2004). The transition to coparenthood: parents’ prebirth expectations and early coparental adjustment at 3 months postpartum. Dev. Psychopathol. 16, 711–733. doi: 10.1017/S0954579404004742, 15605633

[ref32] McHale J. Kuersten-Hogan R. Lauretti A. (2000). “Evaluating coparenting and family-level dynamics during infancy and early childhood: the Coparenting and family rating system,” in Family Observational Coding Systems: Resources for Systemic Research, eds. Kerig P. Lindahl K. (New Jersey: Erlbaum), 151–170.

[ref33] McHale J. Lauretti A. Talbot J. Pouquette C. (2002). “Retrospect and prospect in the psychological study of coparenting and family group process,” in Retrospect and Prospect in the Psychological Study of Families, eds. McHale J. Grolnick W. (New Jersey: Erlbaum), 127–165.

[ref34] McHale J. Tissot H. Mazzoni S. Hedenbro M. Salman-Engin S. Philipp D. A. . (2023). Framing the work: a coparenting model for guiding infant mental health engagement with families. Infant Ment. Health J. John Wiley and Sons Inc 44, 638–650. doi: 10.1002/imhj.22083, 37608513

[ref35] Mensi M. M. Balottin L. Rogantini C. Orlandi M. Galvani M. Figini S. . (2020). Focus on family functioning in anorexia nervosa: new perspectives using the Lausanne Trilogue Play. Psychiatry Res. 288:112968. doi: 10.1016/j.psychres.2020.112968, 32320861

[ref36] Minuchin S. (1974). Families & Family Therapy. Harvard U. Press. Cambridge, Massachusetts: Harvard University Press.

[ref37] Minuchin S. Rosman B. L. Baker L. (1978). Psychosomatic Families: Anorexia Nervosa in Familiar Context. Cambridge, MA: Harvard University Press.

[ref38] Minuchin S. Rosman B. L. Baker L. Liebman R. (2009). Psychosomatic Families: Anorexia Nervosa in Context. Cambridge, Massachusetts: Harvard University Press.

[ref17] Morrill M. I. Hines D. S. Mahmood S. Córdova J. V. (2010). Pathways between marriage and parenting for wives and husbands: the role of coparenting. Fam. Process 49, 59–73. doi: 10.1111/j.1545-5300.2010.01308.x, 20377635 PMC4896491

[ref39] National Institute for Health and Care Excellence (2020). Eating Disorders: Recognition and Treatment. London: National Institute for Health and Care Excellence (NICE). 33689256

[ref40] Philipp D. A. Mazzoni S. Hedenbro M. Tissot H. Darwiche J. Keren M. . (2026). Enhancing coparenting using video feedback: consensus guidelines for infant and preschool families. Fam. Relat. 75, 261–276. doi: 10.1111/fare.70054

[ref41] Polanczyk G. V. (2015). Annual research review: a meta-analysis of the worldwide prevalence of mental disorders in children and adolescents. J. Child Psychol. Psychiatry Allied Discip. 56, 345–365. doi: 10.1111/jcpp.12381, 25649325

[ref9001] Ricci C. Selvini-Palazzoli M. (1984). Interactional Complexity and Communication. Fam. Process 23, 169–176. doi: 10.1111/j.1545-5300.1984.00169.x, 6734789

[ref42] Russell G. F. M. (1987). An evaluation of family therapy in anorexia nervosa and bulimia nervosa. Arch. Gen. Psychiatry 44, 1047–1056. doi: 10.1001/archpsyc.1987.01800240021004, 3318754

[ref43] Sturge-Apple M. L. Davies P. T. Cummings E. M. (2006). Impact of hostility and withdrawal in interparental conflict on parental emotional unavailability and children’s adjustment difficulties. Child Dev. 77, 1623–1641. doi: 10.1111/j.1467-8624.2006.00963.x, 17107450

[ref44] Tafà M. Cerniglia L. Cimino S. Bracaglia F. (2019). Finalmente il coparenting: l’importanza della cogenitorialità per il benessere dei figli. Una ricerca su adolescenti con disturbi alimentari. Psicol. Clin. dello Sviluppo 23, 201–220. doi: 10.1449/94339

[ref45] Teubert D. Pinquart M. (2010) The association between coparenting and child adjustment: a meta-analysis Parenting Taylor & Francis Group 10: 286–307. doi: 10.1080/15295192.2010.492040

[ref46] Zhao F. (2022). The association between coparenting behavior and internalizing/externalizing problems of children and adolescents: a meta-analysis. Int. J. Environ. Res. Public Health 19:10346. doi: 10.3390/ijerph191610346, 36011980 PMC9407961

